# Mitochondrial ROS and the Effectors of the Intrinsic Apoptotic Pathway in Aging Cells: The Discerning Killers!

**DOI:** 10.3389/fgene.2016.00161

**Published:** 2016-09-14

**Authors:** Siegfried Hekimi, Ying Wang, Alycia Noë

**Affiliations:** Department of Biology, McGill UniversityMontreal, QC, Canada

**Keywords:** ROS signaling, superoxide, intrinsic apoptotic pathway, mitochondrial ROS, longevity, aging

## Abstract

It has become clear that mitochondrial reactive oxygen species (mtROS) are not simply villains and mitochondria the hapless targets of their attacks. Rather, it appears that mitochondrial dysfunction itself and the signaling function of mtROS can have positive effects on lifespan, helping to extend longevity. If events in the mitochondria can lead to better cellular homeostasis and better survival of the organism in ways beyond providing ATP and biosynthetic products, we can conjecture that they act on other cellular components through appropriate signaling pathways. We describe recent advances in a variety of species which promoted our understanding of how changes of mtROS generation are part of a system of signaling pathways that emanate from the mitochondria to impact organism lifespan through global changes, including in transcriptional patterns. In unraveling this, many old players in cellular homeostasis were encountered. Among these, maybe most strikingly, is the intrinsic apoptotic signaling pathway, which is the conduit by which at least one class of mtROS exercise their actions in the nematode *Caenorhabditis elegans*. This is a pathway that normally contributes to organismal homeostasis by killing defective or otherwise unwanted cells, and whose various compounds have also been implicated in other cellular processes. However, it was a surprise that that appropriate activation of a cell killing pathway can in fact prolong the lifespan of the organism. In the soma of adult *C. elegans*, all cells are post-mitotic, like many of our neurons and possibly some of our immune cells. These cells cannot simply be killed and replaced when showing signs of dysfunction. Thus, we speculate that it is the ability of the apoptotic pathway to pull together information about the functional and structural integrity of different cellular compartments that is the key property for why this pathway is used to decide when to boost defensive and repair processes in irreplaceable cells. When this process is artificially stimulated in mutants with elevated mtROS generation or with drug treatments it leads to lifespan prolongations beyond the normal lifespan of the organism.

## Reactive Oxygen Species (ROS): Toxicity and Signal Functions

Superoxide ($O_{2}^{•-}$) is generated when an extra electron is donated to molecular oxygen, and hydrogen peroxide (H_2_O_2_) is generated in a variety of ways, including by the action of superoxide dismutases (SODs) on superoxide. Peroxide in turn can be broken down by catalase and other enzymatic processes. The chemical reactivity of reactive oxygen species (ROS) means that they can be *toxic.* For example, excessive ROS can inflict various oxidative damage upon macromolecules (nucleic acids, lipids, and proteins), thus compromising the integrity and viability of the cell. By reacting with lipids in the membrane and inducing lipid peroxidation, ROS can cause structural and functional changes of the plasma and intracellular organelle membranes. Oxidation of protein cysteine (Cys) residues can lead to the formation of sulfenic acid (-SOH), which can form disulfide bonds (S-S) with nearby Cys residues or undergo further oxidation to sulfinic acid (-SO_2_H) or sulfonic acid (-SO_3_H). These and other oxidative modifications to proteins may cause conformational changes, cross-linking, and peptide backbone breakage that ultimately alter protein functionality, including loss of biological activity (Friguet, 2006; Roos and Messens, 2011). In regards to oxidative damage to nucleic acids, ROS most frequently modify bases, inducing strand crosslinking and breakage (Jena, 2012). Guanine is the base most susceptible to oxidation, products of which can mispair with adenine during DNA synthesis yielding G:C to T:A transversions (Perillo et al., 2008). They may also stall DNA replication, resulting in single or double strand breaks (Cheng et al., 1992).

But ROS are also *signaling molecules* that impinge on classic signal transduction pathways, including via irreversible modification of macromolecules (Finkel, 2012; Holmstrom and Finkel, 2014). The best characterized examples of ROS signaling involve the oxidation of cysteine residues in redox-sensitive proteins. These oxidative modifications are reversible by the action of glutaredoxin (GRX) and thioredoxin (TRX), making them ideal for participation in signaling (Reczek and Chandel, 2015). PTEN, a tumor suppressor that regulates cell migration and growth, is a well-characterized example of a redox-sensitive protein. Analysis of cysteine mutants of PTEN revealed that H_2_O_2_ exposure causes Cys71 to form a disulfide bond with the essential Cys124 in the active site, thus inactivating it (Lee et al., 2002). ROS also affect the activation of mitogen-activated protein kinases, like p38 and ERK1/2. Treatment of rat adrenal medulla (PC12) cells with 500 μM peroxynitrite (a strong oxidant resulting from the interaction of superoxide and nitric oxide) increased the phosphorylation levels of both p38 and ERK1/2 within minutes (Jope et al., 2000). ROS also regulate apoptosis and necrosis (Shen and Liu, 2006) and stimulate autophagy (Filomeni et al., 2015). Even more unexpectedly, the modification of DNA via ROS is a requirement for the transcriptional activation of some genes. It has been shown that exposure of cells to estrogen causes the formation of 8-oxoguanine (8-oxoG) which is recognized by the DNA glycosylase OGG1. Excision of the 8-oxoG lesion by OGG1 creates single-strand breaks (SSBs) which then recruits topoisomerase IIβ to estrogen-responsive DNA elements in the promoter region of estrogen-responsive genes. This allows DNA to bend and thereby allows for the promoter to be brought in close proximity with the transcriptional initiation complex (Nathan and Cunningham-Bussel, 2013).

## ROS and Aging

The potential toxicity of ROS, in particular that of ROS originating from mitochondria (mtROS), has led to the formulation of the oxidative stress theory of aging, which suggested that accumulation of oxidative damage to macromolecules is at the heart of the aging process (Ku et al., 1993). Recently, however, we and others have proposed alternative interpretations of some of the observations that led to the formulation of the theory (Blagosklonny, 2008; Lapointe and Hekimi, 2010). For example, we have proposed that ROS damage might not be causally involved in the aging process but that ROS levels are correlated with the aged phenotype because they modulate signal transduction pathways that are specifically involved in responding to the type of cellular stresses that are brought about by aging (Hekimi et al., 2011). In other words, ROS increase with age because they are involved in combating the consequences of aging, not because they cause aging. This hypothesis is supported by observations we review below in a variety of organisms, in particular in *Caenorhabditis elegans.*

## Uncoupling ROS and Aging

If ROS cause aging then ROS generation and detoxification could not be uncoupled from effects on lifespan. But, for example, we and others have been able to show that knockdown and knockout of superoxide dismutases and consequent elevations in oxidative damage are not associated with decreased lifespan (Yang et al., 2007; Doonan et al., 2008; Yen et al., 2009; Van Raamsdonk and Hekimi, 2012), even when all five *C. elegans* SODs are lost simultaneously and the organism has become dramatically hypersensitive to experimental elevation of superoxide (Van Raamsdonk and Hekimi, 2012). In fact, loss of SOD-2 is even associated with increased lifespan (Van Raamsdonk and Hekimi, 2009; see below for further discussion). This uncoupling also works the other way round: elevating superoxide dismutase activity with SOD mimetics does not prolong lifespan (Keaney et al., 2004). In fact, the review of much of the literature about ROS and aging in *C. elegans* does not reveal any tight association between the two (Van Raamsdonk and Hekimi, 2010). Moreover, lifespan extension in some SOD overexpression models is likely unrelated to antioxidant activity (Cabreiro et al., 2011).

## Mitochondrial Function and Aging

In seeking to investigate the association between ROS and aging, or the lack thereof, we found that, although aging was not tightly associated with ROS levels, lifespan could be *lengthened* rather than shortened by conditions and treatments that were generally believed to be deleterious to mitochondria. For example, over 20 years ago, the *C. elegans clk-1* mutants were among the very first long-lived mutants to be described (Wong et al., 1995). *clk-1* was subsequently shown to encode a hydroxylase necessary for ubiquinone (UQ; coenzyme Q, CoQ) biosynthesis (Ewbank et al., 1997) and, as a consequence for normal mitochondrial function (Felkai et al., 1999). Most strikingly in regard to aging, the mitochondria of *clk-1* mutants lose the ability to accumulate an inner membrane potential-dependent dye at a younger age than the wild type despite their lengthened lifespan (Felkai et al., 1999).

In addition, purified *clk-1* mitochondria show elevated superoxide levels and oxidative stress (Yang and Hekimi, 2010a). A subsequent genetic screen for *clk-1*-like mutants uncovered several additional long-lived mutants that, like *clk-1*, displayed a slow phenotype, maternal rescue, low energy metabolism and long life, but not decrease in overall oxidative damage (de Jong et al., 2004; Van Raamsdonk et al., 2010). Interestingly, the screen to identify these mutants was based on their slow phenotype and not their long life. Yet, all mutants identified in this way showed increased lifespan, suggesting that a hypo-metabolic phenotype is sufficient for increased lifespan.

## Long-Lived *C. elegans* Electron Transport Chain (ETC) Mutants

The best studied genes among the *clk-1*-like genes all encode proteins whose activity is broad, affecting several, more or less independent processes: CLK-1 is necessary for UQ synthesis which is present as an electron transporter in most cellular membranes, carrying out diverse and independent functions in different locations (Wang and Hekimi, 2016); CLK-2 affects telomeres in *C. elegans* (Benard et al., 2001) and is a regulator of the stability of PI3K-related protein kinases, including the mammalian target of rapamycin (mTOR; Takai et al., 2007); TPK-1 is necessary for thiamine metabolism and therefore affects the activity of all thiamine-dependent enzymes (de Jong et al., 2004).

To identify mutations whose effects on energy metabolism and ROS were more specific, we screened for further *clk-1*-like mutants but without requiring the presence of a maternal effect (phenotypic rescue of a homozygous mutant animal by a wild type allele in the mother). This yielded two point mutations in subunits of the electron transport chain: *isp-1(qm150)* which alters one amino acid in the vicinity of the iron-sulfur center in the Rieske Iron Sulfur Protein of mitochondrial complex III (Feng et al., 2001), and *nuo-6(qm200)* which affects NDUFB4, a little studied subunit of complex I (Yang and Hekimi, 2010b) that is a preferential target of nitration by peroxynitrite in bovine heart (Murray et al., 2003). Both mutants are very long-lived and their effects on lifespan are not additive: that is, the double mutants are long-lived but live no longer than the single mutants (Yang and Hekimi, 2010b). The study of these mutants yielded the most unequivocal evidence connecting increased generation of mitochondrial ROS (mtROS) to increased lifespan. Indeed, purified mitochondria from both mutants show elevated superoxide generation (Yang and Hekimi, 2010a), their increased lifespan is suppressed by antioxidant treatment (*N*-acetyl cysteine, or vitamin C; Yang and Hekimi, 2010a), and their effect on lifespan can be phenocopied by treatment with very low level (0.1 mM) of the pro-oxidant paraquat (PQ; Yang and Hekimi, 2010a; Hwang et al., 2014).

## The Lessons of PQ

Compounds such as PQ (1,1′-dimethyl-4,4′-bipyridinium dichloride) can accept electrons from an electron carrier in the ETC and transfer them to molecular oxygen to produce superoxide ($O_{2}^{•-}$) and are used to increase cellular, and in particular mitochondrial, oxidative stress (Bus et al., 1976; Cocheme and Murphy, 2008; Robb et al., 2015). In fact, mitochondria are likely the major site of superoxide generation by the action of PQ (Cocheme and Murphy, 2008). More specifically, it is believed that PQ^2+^ is reduced by electrons from mitochondrial complex I to form a radical cation (PQ^•+^), which reacts with O_2_ to produce superoxide.

The study of the effect of PQ was particularly instructive as it was found that it was not additive to the lifespan effect of the *isp-1* and *nuo-6* mutations, although it was additive to the effect of other previously identified long-lived mutants [e.g., *eat-2* (which lives long because of calorie restriction) and *daf-2* (which has reduced insulin-like signaling activity); Yang and Hekimi, 2010a]. In addition, the effect of PQ was not always, or very incompletely, suppressed by a variety of loss-of-function mutations in genes believed to function downstream of previously defined longevity pathways (e.g., *daf-2, aak-2, jnk-1, wwp-1, skn-1, hsf-1, hif-1*; Yang and Hekimi, 2010a).

**FIGURE 1:**
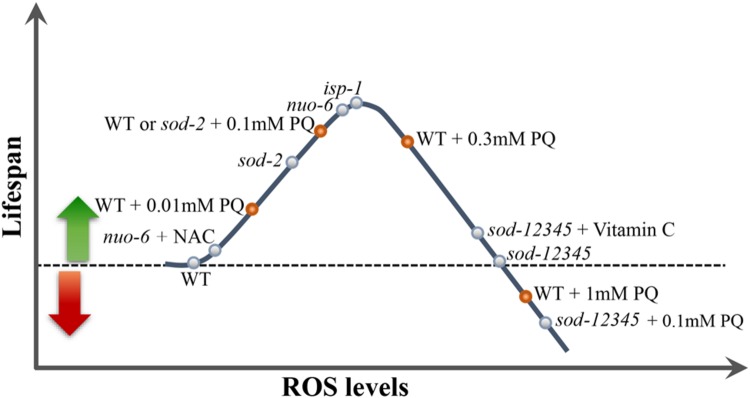
**The inverted U-shaped relationship between ROS and lifespan.** Increasing ROS levels in animals in which ROS levels are lower than the optimum for longevity results in an increase of lifespan. Beyond that optimal level, further increases have less effect and finally decrease animal lifespan when ROS become too elevated. Note that such a relationship implies that both pro-oxidants and antioxidants can increase or decrease lifespan depending on the genotype of the animals, but possibly depending also on the exact cellular or sub-cellular target of any particular redox-active compound. See **Figure 2** for schematic representation of the lifespan data of some of the mutants plotted on the curve.

We determined the dose-response relationship between the concentration of PQ and lifespan of the wild type and found to be in the form of an inverted U-shape, with an optimum concentration of 0.1 mM under the conditions used (Van Raamsdonk and Hekimi, 2012; **Figure 1**). This suggests that increased mitochondrial ROS generation increases lifespan (by signaling mechanisms discussed below), but that the effect becomes gradually weakened at higher concentrations by lifespan-shortening effects due to the toxicity of abnormally high ROS generation. Alternatively, it is possible that the cell type (unknown) or the sub-cellular site (likely mitochondria) where PQ acts preferentially has its optimum effect at a particular concentration of ROS, without implying that slightly higher concentrations necessarily produce unmanageable amounts of additional oxidative damage.

**FIGURE 2:**
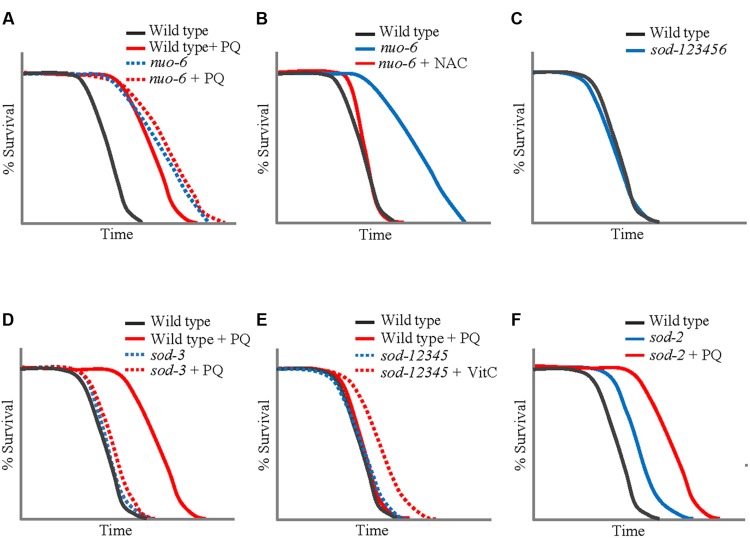
**Lifespans of wild type *Caenorhabditis elegans* and mutants treated with PQ (0.1 mM) or antioxidants. (A)** The PQ treatment has no effect on the lifespan of *nuo-6(qm200)* mutant (Yang and Hekimi, 2010a). **(B)** Antioxidant *N*-acetylcysteine (NAC) fully eliminates the extended longevity of *nuo-6(qm200)* (Yang and Hekimi, 2010a). **(C)** *sod-12345* worms that lack all five *sod* genes live a normal lifespan (Van Raamsdonk and Hekimi, 2012). **(D)** The PQ treatment lengthens the lifespan of the wild type but is without effect on the longevity of *sod-3(tm760)* knockout mutant (Yee et al., 2014). **(E)** The lifespan of *sod-12345* worms can be increased by the treatment with vitamin C (VitC; Van Raamsdonk and Hekimi, 2012). **(F)** Treatment with PQ further increases the lifespan of *sod-2* mutant to that of the wide-type under the same treatment condition (Yang and Hekimi, 2010a). All figures are redrawn from the published work cited.

The idea of an inverted U-shaped relationship between ROS and lifespan suggested by the effects of PQ allowed to conceptualize previous results. For example, it suggests that the level of mitochondrial ROS generation in *isp-1* and *nuo-6* mutants is close to that produced by the optimum concentration of PQ (0.1 mM), explaining why 0.1 mM is not additive to the effect of the mutations, and why treatment with antioxidants shorten lifespan (**Figures 2A,B**). In addition, we tested PQ on a mutant strain (abbreviated *sod-12345*) which lacks all five *C. elegans sod* genes (Van Raamsdonk and Hekimi, 2012). The lifespan of these animals is wild type (**Figure 2C**), but, not surprisingly, they are hypersensitive to PQ and no concentration of PQ is capable of increasing their lifespan. Rather, even the lowest concentrations are lifespan-shortening. This could be due to the toxicity of excessive superoxide in the absence of detoxification. Alternatively, it is generally believed that peroxide (which results from the action of SODs on superoxide) is the main intracellular ROS messenger, due to its greater stability and capacity to cross membranes (Holmstrom and Finkel, 2014). Thus the absence of adequate peroxide generation because of the absence of SODs could shorten lifespan by preventing normal, beneficial, ROS signaling. This possibility is reinforced by the finding that the lifespan increase produced by the optimum concentration of PQ (0.1 mM) is abolished by loss of SOD-3 (**Figure 2D**), the minor mitochondrial SOD, but not by loss of SOD-2 (Yee et al., 2014). Note that the two hypotheses (toxicity of excessive superoxide and insufficient peroxide signaling) are not mutually exclusive to explain the lack of beneficial effect of PQ on *sod-12345* mutants.

The wild type lifespan of the *sod-12345* mutant strain allowed for a further test of the inverted U-shaped relationship between ROS and lifespan. As mentioned, these mutants have a normal lifespan that cannot by lengthened by PQ treatment. This suggest that the mutants’ ROS levels produce an exact balance between beneficial effects and toxicity. In other words, the mutants live on the far side of the ∩ (**Figure 1**). We treated these mutants and the wild type with the antioxidant vitamin C (VitC) as well as with Mn^++^, the co-factor of MnSODs, which has superoxide dismutase activity by itself (Archibald and Fridovich, 1981). Both treatment lengthened the lifespan of the mutant strain but were without effect on the wild type (Van Raamsdonk and Hekimi, 2012). This created a striking situation in which a strain devoid of superoxide detoxification lives *longer* than the wild type under the same conditions (**Figure 2E**).

The inverted U-shaped relationship of PQ and lifespan has been used to identify and characterize other activities that might modulate the lifespan effects of ROS. In particular, the activities of AMP kinase and HIF-1 have been found to be able to alter the placement of the ∩ along both the lifespan and dosage axes (Hwang et al., 2014). This suggests that there might be complex feedback mechanisms which respond to change of intracellular ROS levels and, via an additional level of ROS modulation, regulate downstream effector pathways and ultimately survival. PQ treatment also allows for testing of the involvement of beneficial ROS signaling in the long lifespan of *clk-1* and *sod-2* mutants (**Figure 2F**). Indeed, *isp-1;clk-1* and *isp-1;sod-2* double mutants are lethal or very sick, respectively (Van Raamsdonk and Hekimi, 2009), which makes them poor tools. However, we found that the action of PQ on lifespan was additive to the effect of *sod-2* and *clk-1* mutations (in contrast to the lack of additivity on *isp-1* and *nuo-6*). How *clk-1* affects lifespan in *C. elegans* is not well-understood and thus might not involve beneficial ROS signaling, even if an alteration in ROS metabolism is part of the phenotype of these mutants. However, it is difficult to envision that the action of *sod-2* mutations would not be via an increase in mitochondrial superoxide generation. This suggests that there might be more than one mechanisms of lifespan extension that involves mitochondrial ROS generation (see below the discussion of the apoptotic pathway signaling for additional evidence for this). Furthermore, by the logic of additivity, there might be three distinct mechanisms as *clk-1* and *sod-2* are in fact additive (Van Raamsdonk and Hekimi, 2009). Although, as for *sod-2* mutants, there is no direct proof that the altered ROS metabolism of *clk-1* mutants is responsible for their longevity.

An number of additional studies have also been exploring the role of ROS in mitochondrial longevity, and a number of additional regulatory molecules, in particular transcription factors, have been implicated in parts of the response of *C. elegans* to increased ROS generation, including following PQ treatment (An and Blackwell, 2003; Curtis et al., 2006; Lee et al., 2010; Paek et al., 2012; Baruah et al., 2014; Hwang et al., 2014). These include CEP-1(p53), SKN-1(Nrf-1/2), and DAF-16(FOXO). They all appear to function downstream of the mtROS signal and none is absolutely required for the longevity response (Yang and Hekimi, 2010a; Hwang et al., 2014). SKN-1 and DAF-16 in particular appear to be important for resistance to ROS damage (Honda and Honda, 1999; An and Blackwell, 2003).

## Lifespan Increase by Knockdown of ETC Subunits Is Fully Distinct From ROS-Dependent Signaling

A number of studies including several genome-wide screening experiments have demonstrated that RNAi knockdown of ETC subunits or components of the mitochondrial translation machinery is sufficient to extend lifespan (Dillin et al., 2002; Lee et al., 2003; Hamilton et al., 2005; Chen et al., 2007; Curran and Ruvkun, 2007; Rea et al., 2007; Copeland et al., 2009; Yang and Hekimi, 2010b; Durieux et al., 2011; Houtkooper et al., 2013). In *C. elegans*, the most robust effect was seen with RNAi clones against the ETC genes *nuo-2, nuo-6*, *isp-1*, *cyc-1, cco-1*, and *apt-3.* An intriguing finding involving RNAi experiments is that at least for some ETC genes, e.g., *isp-1* and *nuo-6*, the mechanism behind the effect of RNAi knockdowns is distinct from the mechanism that allows the point mutants to live a markedly extended lifespan. First, for both *nuo-6* and *isp-1* genes, when the mutant strain was treated with the RNAi against the other gene, lifespan was further lengthened, resulting in dramatically long lifespans (Yang and Hekimi, 2010b). These additive interactions suggest that the allele and RNAi treatment involved affect survival via separate downstream mechanisms. Another possible explanation would be that different interventions at the two distinct sites (for example, *isp-1* mutation and *nuo-6* RNAi) act via the same mechanism and in combination they act additively simply because each one by itself has a submaximal effect. However, we consider this unlikely, since more severe impairment of ETC induced by either a higher dose of RNAi or double mutations failed to achieve more prolonged lifespans (Yang and Hekimi, 2010b). Moreover, consistent with different mechanisms of action, the phenotypes of *isp-1(qm150)* and *nuo-6(qm200)* mutants and those of RNAi knockdown were found to be distinct in almost every ways, regarding oxygen consumption and ATP levels, patterns of growth and fertility, behavioral rates, resistance to paraquat, autophagy, as well as expression levels of SODs and other gene expression markers (Yang and Hekimi, 2010b).

Thus, it appears that, although genetic mutations and RNAi knockdown of specific ETC subunits extend lifespan in worms, this is by totally different mechanisms. As will be brought up further in a section below, ETC gene mutations act via the effect of altered mtROS levels on apoptotic signaling, while the longevity of RNAi-treated worms is not sensitive to loss of apoptotic signaling (Yee et al., 2014). Finally, in this regard, it has been proposed that induction of mitochondrial unfolded protein response (UPR^mt^) promotes longevity and is directly responsible for the lifespan extension from ETC inhibition (Yang and Hekimi, 2010b; Durieux et al., 2011). However, the results have since become controversial (Pujol et al., 2013; Rauthan et al., 2013; Bennett et al., 2014). More recently, it was discovered that knockdown of the complex IV subunit 1 (*cco-1*) is associated with chromatin restructuring that is required for the full activation of UPR^mt^ and is partially accountable for the lifespan-extending effect (Tian et al., 2016). Whether, other types of ETC alternations induce such changes is yet to be seen.

## Other Pro-Longevity Effects of ROS, Including Evidence From Different Types of Organisms

Mitochondria have been long considered to be important for the life-extending effect of dietary restriction (DR). It was initially proposed that DR induces a hypo-metabolic state and in turn lowers the rate of mtROS production and decreases ROS damage which is what extends lifespan (Sohal and Weindruch, 1996; Sohal et al., 2009). However, how DR affects metabolic rate and mitochondrial physiology is still highly controversial with many studies showing mixed findings (Sohal et al., 1994; Gredilla et al., 2001; Lambert et al., 2004). Specifically, several more recent studies have reported that at least some DR regimens do not suppress the rate of mitochondrial respiration, but instead increases it (Lin et al., 2002; Nisoli et al., 2005; Bishop and Guarente, 2007; Zid et al., 2009). In a yeast model of growth in low glucose a metabolic shift toward increased respiration was observed, and inhibition of ETC function severely attenuated lifespan increase (Lin et al., 2002). Similar findings were also demonstrated in the fruit fly (Zid et al., 2009; Bahadorani et al., 2010). Furthermore, in some rodent DR models, an induction in mitochondrial biogenesis was described (Nisoli et al., 2005; Lopez-Lluch et al., 2006; Lanza et al., 2012). In line with the growing recognition of ROS as signaling molecules, it has been proposed that DR promotes an increase in intracellular ROS that activates a program of responses to stress, thereby leading to prolonged lifespan. Additional evidence came from the findings that, under a particular DR regimen (imposed by glycolysis inhibitor 2-deoxy-D-glucose), worms show overall enhanced respiration rate and increased overall ROS formation (measured indirectly by DCF fluorescence) and that upon treatment with antioxidants the lifespan increase in the presence of 2-deoxy-D-glucose was diminished (Schulz et al., 2007). However, it is of note that no elevation of mtROS was detected in *eat-2* mutants, a widely acknowledged genetic mimic of DR. This, added to the observation that PQ can further extend the lifespan of *eat-2* mutants (Yang and Hekimi, 2010a), suggests that at least some DR models do not involve elevated ROS.

Reduced insulin/IGF-1-like signaling (IIS) also prolong lifespan in multiple different species (Kenyon, 2005). In *C. elegans*, the IIS pathway is activated through DAF-2, the solo insulin/IGF-1 receptor. It ultimately down-regulates DAF-16, a FOXO transcription factor, which regulates a wide variety of genes associated with growth, stress resistance, metabolism and survival (Ogg et al., 1997). Zarse et al. (2012) reported that *daf-2* mutants have increased mitochondrial metabolism compared to the wild type but reduced overall ROS levels. On the other hand, the same study showed an early transient increase of ROS levels following *daf-2* RNAi in adult worms (Zarse et al., 2012). The importance of this transient ROS increase was further underlined by the observation that inhibition of this ROS signal eliminated a part of the lifespan extension afforded by *daf-2* RNAi (Zarse et al., 2012). Furthermore, it is of note that elevated superoxide production (but not overall ROS levels) was detected in *daf-2* mutants (Yang and Hekimi, 2010a) and exogenous antioxidants have a shortening effect on their lifespan (Yang and Hekimi, 2010a; Zarse et al., 2012), also indicating that some of the mutant longevity phenotype is mediated by a ROS-dependent mechanism.

Lastly, such observations are not limited to *C. elegans*. There is growing data suggesting mtROS signaling can favor longevity in other organisms (**Table 1**). Decreased mTOR signaling has been demonstrated to extend lifespan in multiple organisms, the mechanisms of which appear to be exceedingly complex and are not fully understood (Johnson et al., 2013). Among them, a role of mtROS downstream of mTOR signaling was implied by the findings that reduced mTOR signaling in the yeast *tor1Δ* mutants enhanced mitochondrial respiration and mtROS generation during growth and that diminishing mtROS levels in the mutants significantly eliminated extended chronological lifespan (CLS; Bonawitz et al., 2007; Pan et al., 2011). Furthermore, deletion of *sod2* or treating a wild type strain during growth with the superoxide generator menadione was shown to be sufficient to lengthen CLS, furthering support for the conclusion of a role for mtROS as a key mediator of yeast CLS extension (Pan et al., 2011; Schroeder et al., 2013). The group further searched for factors that are required for the observed CLS extension resulting from menadione-generated ROS. Intriguingly, they identified activation of the Tel1p and Rad53p DNA-damage response kinases and the downstream Rph1p-regulated subtelomeric silencing as a key outcome of mtROS signaling that ultimately prolongs the survival in stationary phase (i.e., extension of CLS; Schroeder et al., 2013).

**Table 1:** Lifespan effect of mutations and treatments that affect ROS metabolism.

Organism	Genetic mutants or pharmacological intervention	Direct target and effects	Effect on ROS levels	Effect on lifespan	Reference
**Yeast**	*Tor1Δ*	mTOR ↓	MtROS ↑	CSL ↑	Bonawitz et al., 2007; Pan et al., 2011
	Menadione	$O_{2}^{•-}$ generation ↑	MtROS ↑	CSL ↑	Pan et al., 2011
***Caenorhabditis elegans***	*nuo-6(qm200)*	ETC complex I ↓	MtROS ↑	↑	Yang and Hekimi, 2010a,b
	*isp-1(qm150)*	ETC complex III ↓	MtROS ↑	↑	Feng et al., 2001; Yang and Hekimi, 2010a
	*clk-1(qm30)*	Ubiquinone biosynthesis ↓	ROS ↑	↑	Wong et al., 1995; Yang and Hekimi, 2010a
	Paraquat	$O_{2}^{•-}$ generation ↑	MtROS ↑	↑	Lee et al., 2010; Yang and Hekimi, 2010a
	*sod-2*	Mito. $O_{2}^{•-}$ degradation ↓	MtROS ↑	↑	Van Raamsdonk and Hekimi, 2009
	*sod-1*	Cyto. $O_{2}^{•-}$ degradation ↓	NK	↔	Van Raamsdonk and Hekimi, 2009
	Rotenone, piericidin A	ETC complex I ↓	NK	↑	Schmeisser et al., 2013a
	2-deoxyglucose	Glycolysis inhibition, DR	ROS ↑	↑	Schulz et al., 2007
	Arsenite	Toxin	ROS ↑	↑	Schmeisser et al., 2013b
**Fruit fly**	NDUFS1/ND75	ETC complex I ↓	ROS ↑	↑	Owusu-Ansah et al., 2013
	*Sod1*^-^*^/^*^-^	Cyto. $O_{2}^{•-}$ degradation ↓	NK	↓	Phillips et al., 1989
	*Sod2*^-^*^/^*^-^	Mito. $O_{2}^{•-}$ degradation ↓	NK	↓	Duttaroy et al., 2003
	SOD1 overexpression	Cyto. $O_{2}^{•-}$ degradation ↑	NK	↑	Parkes et al., 1998
	SOD2 overexpression	Mito. $O_{2}^{•-}$ degradation ↑	NK	↑	Sun et al., 2002
	MCAT overexpression	Mito. H_2_O_2_ degradation ↑	NK	↔	Mockett et al., 2003
**Mice**	*Mclk1^+/^*^-^	Ubiquinone biosynthesis↓	MtROS ↑	↑	Liu et al., 2005; Lapointe and Hekimi, 2008; Lapointe et al., 2009
	*Surf1*^-^*^/^*^-^	ETC complex IV↓	↔	↑	Dell’agnello et al., 2007; Pulliam et al., 2014
	*Sod1*^-^*^/^*^-^	Cyto. $O_{2}^{•-}$ degradation ↓	Damage ↑	↓	Elchuri et al., 2005
	*Sod2*^-^*^/^*^-^	Mito. $O_{2}^{•-}$ degradation ↓	Damage ↑	↓	Li et al., 1995
	*Sod1^+/^*^-^	Cyto. $O_{2}^{•-}$ degradation ↓	Damage ↑	↔	Elchuri et al., 2005
	*Sod2^+/^*^-^	Mito. $O_{2}^{•-}$ degradation ↓	Damage ↑	↔	Van Remmen et al., 2003
	SOD1 overexpression	Cyto. $O_{2}^{•-}$ degradation ↑	NK	↔	Huang et al., 2000; Perez et al., 2009
	SOD2 overexpression	Mito. $O_{2}^{•-}$ degradation ↑	NK	↔	Jang et al., 2009; Perez et al., 2009
	MCAT overexpression	Mito. H_2_O_2_ degradation ↑	H_2_O_2_ ↓	↑	Schriner et al., 2005

$O_{2}^{•-}$, superoxide; Mito., mitochondrial; DR, dietary restriction; CLS, chronological lifespan; Cyto., cytoplasmic; NK, not known; ↔, no change.

In metazoans such as fruit flies and mice, mutations and strong knockdown of ETC subunits or other essential mitochondria components are embryonic lethal or severely limit lifespan (Larsson et al., 1998; Trifunovic et al., 2004; Kujoth et al., 2005; Liu et al., 2007; Vempati et al., 2008; Hughes and Hekimi, 2011; Burman et al., 2014). However, there are a few studies that do suggest a possible benefit of high mtROS. In fruit flies, mild knockdown of muscle NDUFS1/ND75, a component of ETC complex I, increases ROS levels and prolongs lifespan (Owusu-Ansah et al., 2013). And, the effect on longevity can be abrogated by overexpression of an antioxidant enzyme (catalase or glutathione peroxidase 1), indicating a ROS-dependent mechanism (Owusu-Ansah et al., 2013). Heterozygous deletion of the gene *Mclk1* (mouse *clk-1*) or homozygous knockout of the cytochrome *c* assembly factor *Surf1* extends lifespan in mice (Liu et al., 2005; Dell’agnello et al., 2007; Lapointe et al., 2009). *Mclk1^+/-^* heterozygous mice have a mild UQ deficiency in the mitochondrial inner membrane associated with increased mitochondrial oxidative stress (Lapointe and Hekimi, 2008). They also exhibit a higher HIF-1α level, elevated expression of inflammatory cytokines, and an enhanced immune response (Wang et al., 2010, 2012). HIF-1α is an important regulator of immune and inflammatory responses and is known to be stabilized by mtROS (Chandel et al., 2000). Conceivably, high mtROS in the mutant may positively modulate some cellular responses, such as activation of HIF-1α, which would benefit survival and account for at least a part of the longevity phenotype. On the other hand, no change of mitochondrial ROS production was detected in isolated mitochondria from *Surf1^-/-^* mice (Pulliam et al., 2014). Some of the proteins involved in UPR^mt^ were found to be elevated in muscles, which was postulated to be a possible mechanism for the observed pro-longevity effect (Pulliam et al., 2014).

## The Beneficial Effects of mtROS on Lifespan Are Not Hormetic

To explain beneficial effects of a small increase in ROS levels, it has been suggested that it could result from an increase in ROS defenses in response to ROS-dependent damage, a mechanism sometimes called mito*hormesis* (Ristow, 2014; Yun and Finkel, 2014). To test this, we have examined patterns of gene expression linked to mtROS-dependent longevity. The effect on lifespan of the *isp-1* and *nuo-6* are not additive (Yang and Hekimi, 2010b), nor is the effect of PQ on either mutant (Yang and Hekimi, 2010a). We reasoned that changes in gene expression that are common to both mutants and PQ treatment of the wild type should reveal genes that participate in implementing ROS-dependent lifespan extension. Using Affimetrix gene arrays we observed a great amount of overlap between the effect of each of the three conditions (*isp-1, nuo-6*, and PQ treatment) compared to the untreated wild type (Yee et al., 2014). However, no pattern pointing to a particular mechanism of longevity was obvious in the list of altered genes. There was also no indication of an upregulation of ROS detoxification or repair of oxidative damage, whether considering the genes whose expression is altered in all three conditions, or even when considering all genes changed in either of the three conditions. Please note that the very idea that hormesis is the basis of ROS-dependent longevity is somewhat paradoxical (or circular). Indeed, it implies that ROS cause aging, as this is a necessary premise if one wants to argue that increased protection from ROS (in response to ROS) increases lifespan. Rather we believe our observations indicate that constitutively increased mtROS generation leads to increased longevity because general mechanisms of stress resistance and damage repair are triggered and amplified in the absence of actual damage or stress.

## One of the Mitochondrial ROS Signals Acts Through the Intrinsic Apoptosis Pathway

Above we have presented ample evidence that suggests that mtROS signaling can increase lifespan, possibly by more than one pathway. One signaling pathway that is known to include mtROS sensors is the mitochondrial intrinsic apoptosis signaling pathway (Circu and Aw, 2010). The pathway is called ‘intrinsic’ in contradistinction to the ‘extrinsic’ apoptotic pathway which involves interactions with other cells and receptors at the plasma membrane. Apoptosis *per se* is a highly controlled process that in vertebrates is sensitive to mitochondrial function, including mtROS, via the intrinsic pathway (Wang and Youle, 2009). In *C. elegans* a number of programmed apoptotic cell deaths occur during development. Defects in apoptosis are scored by counting apoptotic nuclei in embryos or by counting supernumerary neurons in the pharynx (the feeding organ, which has only few neurons). The intrinsic apoptotic machinery of *C. elegans* consists of the BH3-only protein EGL-1 (BH3 stands for Bcl2 homology domain 3), CED-9 (Bcl2-homolog), CED-4 (Apaf1-homolog), and CED-3 (Casp9-homolog). CED-9 is tethered to the outer mitochondrial membrane and is believed to bind CED-4. Upon interaction with EGL-1, CED-9 undergoes structural rearrangements leading to the release of CED-4, which re-localizes to perinuclear membranes where it assembles into active apoptosomes that activate the caspase CED-3. However, in contrast to vertebrates, there is no evidence for any role for mtROS or cytochrome *c* release in regulating apoptosis in *C. elegans*.

Interestingly, the individual proteins of the apoptotic signaling machinery appear to be able to carry out apoptosis-independent functions. For example, EGL-1 and CED-9 affect mitochondrial dynamics (Lu et al., 2011), CED-4 and CED-3 promote neuronal regeneration (Pinan-Lucarre et al., 2012), CED-4 appears to be involved in hypoxic pre-conditioning (Dasgupta et al., 2007) and S-phase checkpoint regulation (Zermati et al., 2007). These and similar findings in other organisms (Galluzzi et al., 2008; Dick and Megeney, 2013) suggest that the individual proteins of the intrinsic apoptotic pathway have bona fide signal transduction activities in other processes.

In fact, we recently provided evidence that mtROS act by signaling through the apoptotic pathway (Yee et al., 2014). Our study showed that mutations in the core apoptosis signaling pathway, which each abolishes apoptosis *per se*, also suppress the longevity of *isp-1* and *nuo-6* mutants, and that induced by PQ. However, these suppressions are entirely independent of apoptosis (meaning actual cell death) as they are independent of the BH3-only protein EGL-1, which is required for all apoptosis in *C. elegans* (Nehme and Conradt, 2008). In contrast, the mutants’ lifespan is suppressed by loss of CED-13, the only other *C. elegans* BH3-only protein and which is not required for apoptosis (Schumacher et al., 2005). In vertebrates, BH3-only proteins have been shown to be sensors of the cellular dysfunctions that trigger apoptotic programs (Galluzzi et al., 2014). We hypothesize that CED-13 similarly senses dysfunctional cellular states to trigger an appropriate stress response. Interestingly, treatment with PQ can bypass the need for CED-13, suggesting that mitochondrial ROS activates the pathway directly, possibly by acting on CED-9, which is physically associated with mitochondria. The epistatic relationships hold very clearly. For example, although PQ does not affect the lifespan of *isp-1* mutants, the shortened lifespan of *isp-1;ced-13* double mutants is lengthened back to the longevity of *isp-1* by treatment with PQ (Yee et al., 2014).

## Specificity of the mtROS-Dependent Apoptotic Signaling Pathway of Longevity

The longevity suppression by loss of the core apoptotic machinery provides an additional tool to characterize the specificity of the mechanisms of ROS signaling under study. First, loss of *ced-4* did not suppress the longevity of *clk-1* and *sod-2*, confirming that there seems to be more than one mtROS-dependent pathway (see above). Furthermore, loss of *ced-4* also does not suppress the longevity of mutants in *daf-2*, which encodes an insulin receptor-like protein, and in the longevity of which mtROS have also been implicated (Zarse et al., 2012). Finally, loss of *ced-4* also does not suppress mutants that characterize other pathways, including *eat-2* for calorie restriction (Lakowski and Hekimi, 1998), and *glp-1* for germline signaling (Hsin and Kenyon, 1999; Berman and Kenyon, 2006).

Interestingly, in addition to increased longevity, the mitochondrial mutants also display a slowing of processes that consume energy, such as development and behavior. Loss of apoptotic signaling suppresses these phenotypes with the same epistatic patterns as for longevity, but it does not suppress the primary defects of low oxygen consumption and low ATP levels. This suggests that ATP-consuming processes are actively inhibited by mtROS signaling, possibly to protect from mitochondrial dysfunction and/or because ATP is diverted to protective processes. Unfortunately, the study of gene expression did not reveal what these processes are, which remains to be determined.

## Relevance to Human Health and Human Aging

**FIGURE 3:**
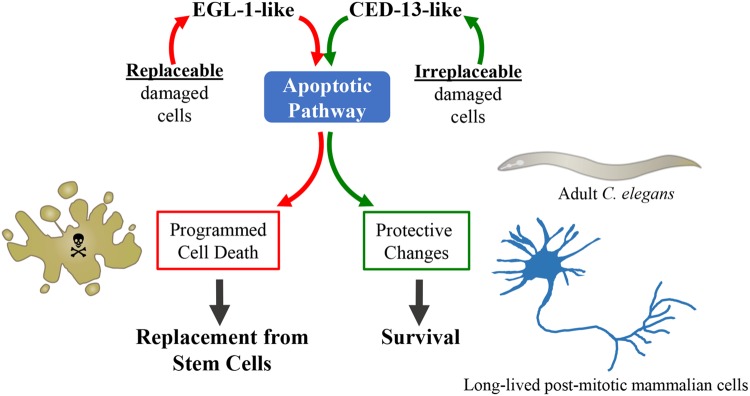
**Non-apoptotic protective function of the intrinsic apoptotic pathway.** In vertebrates, a key function of the apoptotic pathway is to eliminate unneeded, damaged or potentially dangerous cells. Removal of damaged cells by apoptosis followed by normal cell replacement from stem cells is essential for the maintenance of tissue homeostasis. The adult *C. elegans* is post-mitotic and has very few cells, with each cell having a unique function. Thus, the function of the apoptotic pathway in the soma of adult worm cannot be elimination of damaged cells, which, damaged or not, are irreplaceable. One interpretation of the pathway we have uncovered is therefore that *C. elegans* uses the apoptotic pathway under the control of CED-13 to judge the state of disrepair of a cell, not in order to decide to kill it or not, but to determine how strongly to turn on protective changes that help to preserve cellular and, consequently, organismal viability. It is exciting to speculate that there might be a CED-13-like pathway in vertebrate post-mitotic cells such as neurons, cardiomyocytes, or memory T-cells, where such a pathway could be harnessed to stimulate non-apoptotic protective processes.

In a post-mitotic organism such as *C. elegans* damaged somatic cells cannot simply be removed as there is no mechanism to replace them. Instead, the involvement of the apoptotic pathway in longevity suggests that the ability of this pathway to sense cellular dysfunction is used to stimulate a program of stress resistance and repair rather than of cell death. When the pathway is triggered in the absence of damage, such as in the mitochondrial mutants or following low level PQ treatment, its activity results in increased lifespan. In vertebrates there are also cells that cannot simply be replaced when they are injured. Neurons present a dramatic example, as they have very limited regenerative capacities, if any, and neuronal death in both the brain and the periphery results in cognitive and motor impairments. Thus the organism must use other mechanisms than simple apoptotic elimination to ensure that the neurons that sustain molecular injury survive and carry out their functions. Such mechanisms might be more costly than removal of damaged cells followed by replacement from stem cells (or more dangerous, if potentially cancerous cells survive), which is why it is self-destruction and renewal that is commonly used for most cell types. We predict that in vertebrates, as in nematodes, the ability of the apoptotic pathway to sense and integrate cellular dysfunction (Galluzzi et al., 2014) is used to trigger mechanisms of stress-resistance and repair in cells that are difficult to regenerate or are irreplaceable (**Figure 3**). Hopefully, it will be possible to harness such a pathway to promote health and combat age-dependent diseases.

## Author Contributions

All authors listed, have made substantial, direct and intellectual contribution to the work, and approved it for publication.

## Conflict of Interest Statement

The authors declare that the research was conducted in the absence of any commercial or financial relationships that could be construed as a potential conflict of interest.
